# Label-Free Aptamer–Silver Nanoparticles Abs Biosensor for Detecting Hg^2+^

**DOI:** 10.3390/molecules30244785

**Published:** 2025-12-15

**Authors:** Haolin Wang, Xingan Liang, Lan Ye, Licong Fu, Zhiliang Jiang, Dongmiao Qin

**Affiliations:** 1 College of Environment and Food Engineering, Liuzhou Polytechnic University, Liuzhou 545006, China; 2 Key Laboratory of Ecology of Rare and Endangered Species and Environmental Protection, Guangxi Normal University, Ministry of Education, Guilin 541004, China

**Keywords:** mercury ion, AgNPs, label-free aptamer, Abs

## Abstract

In this work, a stable silver nanoparticle (AgNPs) with strong surface plasmon resonance absorption (Abs) signals was synthesized using light-wave technology. In the absence of aptamers, AgNPs can aggregate in a given concentration of salt solution, resulting in significant changes in color. After adding the aptamer (Apt), it was observed that the aptamer can coordinate with AgNPs and adsorb on the surface of AgNPs, thereby maintaining the stability of the nanosol. In the presence of mercury ions (Hg^2+^), their high-affinity reaction with the aptamer compromised the latter’s protective effect on AgNPs, causing the color of the system to change again. Based on this, a simple and rapid new Abs method for detecting Hg^2+^ can be constructed. The linear range was 2.5 × 10^−3^–10.00 μmol/L, and the detection limit (DL) of the system was 2.03 nmol/L.

## 1. Introduction

Nanoparticles (NPs) are widely used in medicine, food safety, environmental governance, and other fields due to their excellent physicochemical attributes, such as conductivity, adjustable light absorption, and high stability [1]. Metal nanoparticles (MNPs) have attracted widespread attention among NPs due to their superior and unique physical and chemical properties. Common MNPs mainly include gold, silver, palladium, platinum, etc. [2]. AgNPs are among the most extensively utilized metal nanoparticles, which have excellent properties such as chemical stability, excellent conductivity, and catalytic activity. The most important characteristics of AgNPs are their antibacterial and anti-inflammatory activities, which enable their application in composite fibers, low-temperature superconducting materials, cosmetics, food industry, agriculture, and electronic components [3]. Yang et al. used tea polyphenols as a reducing agent to synthesize silver nanoparticles by the redox method and immobilized them in sodium alginate (SA) composite film to prepare films that can be used to enhance food preservation ability [4]. At present, physical reduction, chemical reduction, and biological reduction are employed as the main methods to prepare AgNPs [5]. Mishra et al. synthesized AgNPs using the extract of Oscimum Sanctum, and the nanoparticles were used to construct an electrochemical sensor for detecting hydrogen peroxide [6]. Due to the special light absorption properties of AgNPs, their spectral characteristics have been widely studied, such as being used as colorimetric probes [7] and Raman matrices [8]. Vinayagam et al. synthesized gold nanoparticles (AuNPs) using seaweed extract as the reactant, and they established a sensor for hydrogen peroxide by utilizing the color change caused by the oxidation of the nanosol by hydrogen peroxide (H_2_O_2_) [9]. However, the selectivity of the sensor constructed using the oxidation effect of H_2_O_2_ is not very good. Therefore, some specific reactions were introduced to improve the specificity of the method, such as aptamer reaction, antigen–antibody reaction, etc. Aptamers are widely used due to their excellent properties [10], such as high stability, high selectivity, and low cost. Nguyen et al. used the amoxicillin (AMO) aptamer–AMO-specific reaction to regulate the aggregation of gold nanosols induced by Tris-HCl to achieve colorimetric detection of AMO [11]. In this manuscript, stable nanosilver sols were prepared using the light-wave method.

The COVID-19 epidemic has accelerated the development of point-of-care testing (POCT) technology, and various sensor technologies have developed rapidly, such as chemiluminescent sensors [12], fluorescent sensors [13], and colorimetric sensors [14]. Among them, colorimetric sensors have incomparable advantages due to their low cost, simple operation, and intuitive readings, especially in remote and backward areas [15]. Su et al. [16] used the molecular imprinting polymer (MIP)–ketoprofen (KP) reaction to regulate the activity of MIPs@Fe_3_O_4_-Cu enzyme mimics and achieved the detection of KP. Although colorimetric methods (Abs) have unparalleled visual advantages, their sensitivity has always been a problem to be solved. Therefore, it is necessary to develop a series of simple and sensitive Abs detection methods. Mercury (Hg) is a common and highly toxic environmental pollutant produced in human daily life activities. Due to its accumulation in the environment, it has become a significant risk to wildlife and human health [17]. And mercury ions (Hg^2+^) are not metabolized; they can accumulate in the body and cause a variety of diseases, such as kidney and liver disease [18]. In addition, studies have shown that it is associated with certain neurological diseases and can cause rapid toxicity to fetuses and infants [19]. Therefore, it is particularly important to establish an effective and intuitive detection method. At present, many detection methods have been developed, of which the most common are chromatography [20], electrochemical methods [21], atomic absorption methods [22], surface-enhanced Raman scattering (SERS) [23], Abs [24], and fluorescence methods [25]. The chromatographic method, atomic absorption method, electrochemical method, and SERS method have high sensitivity but require expensive analytical instruments and complex operation. Although fluorescence and UV-visible spectrophotometry are convenient and fast, they have low sensitivity. None of the currently reported technologies are able to detect Hg^2+^ quickly, easily, and sensitively. At the same time, analytical methods utilizing aptamers and silver nanoparticles, such as colorimetric and surface-enhanced Raman scattering (SERS) techniques, have been reported. However, the reported colorimetric methods demonstrate lower sensitivity and a narrower linear range (25–500 nM) compared to the colorimetric method developed in this manuscript [26]. Although the SERS method offers high sensitivity (10^−11^ to 10^−6^ M), its operation is relatively complex [27]. In this study, AgNPs with strong Abs signals were prepared by the light-wave method. By integrating these AgNPs with aptamers, we developed an analytical method that exhibits high sensitivity and a wide linear range (2.5 × 10^−3^–10.00 μmol/L). Additionally, the use of Tris-HCl as a buffer solution ensures relatively stable pH conditions, effectively counteracting the influence of small amounts of external strong acids, strong bases, or dilution—thereby enhancing the method’s applicability.

## 2. Results and Discussion

### 2.1. Analytical Principle

**Figure 1:**
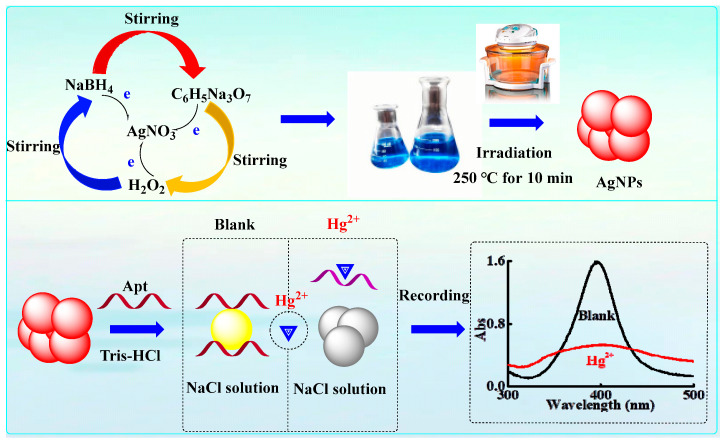
Schematic diagram of the Abs method for detecting Hg^2+^ based on label-free aptamer-AgNPs.

Mixing AgNPs with the Tris-HCl solution (pH 7.85) triggers a color transition from orange-red to yellow, likely due to pH-mediated enhanced dispersion stability of AgNPs, accompanied by a shift in the surface plasmon resonance (SPR) absorption peak [28,29]. Under the effect of the electrostatic shielding effect, the AgNPs-Tris-HCl solution will aggregate in a salt solution of a given concentration. At this time, the color of the nanosol will change. When the aptamer (Apt_Hg_) is added, Apt_Hg_ non-specifically adsorbs onto the surface of AgNPs, thereby inhibiting salt-induced aggregation and preserving the intrinsic color of silver nanoparticles [30]. The adsorption of Apt_Hg_ onto the surface of AgNPs occurs spontaneously, primarily driven by chemical interactions between nucleobases and metal components. Specifically, AgNPs can engage with the nitrogen atoms within the base rings of the aptamer [31]. When the target (Hg^2+^) is added, the aptamer can bind to Hg^2+^ with high affinity and form a stable complex structure (Apt-Hg^2+^). At this time, the structural alteration of the aptamer prevents its adsorption onto silver nanoparticles, thereby triggering salt-induced aggregation and changing the Abs signals [32]. Therefore, a novel Abs methodology for the efficient and expeditious detection of Hg^2+^ was established (Figure 1).

In this study, the speciation of mercury ions differs from that in pure aqueous solutions. The experiment was conducted in a Tris-HCl (pH 7.85) buffer solution and a 7.50 mmol/L NaCl solution. Under these conditions, mercury ions are expected to exist as a mixture of Hg(II) complexes (chloro and hydroxo species) rather than as free aquated Hg^2+^. It is important to note that the speciation is condition-dependent and may vary with changes in experimental conditions.

### 2.2. TEM

**Figure 2:**
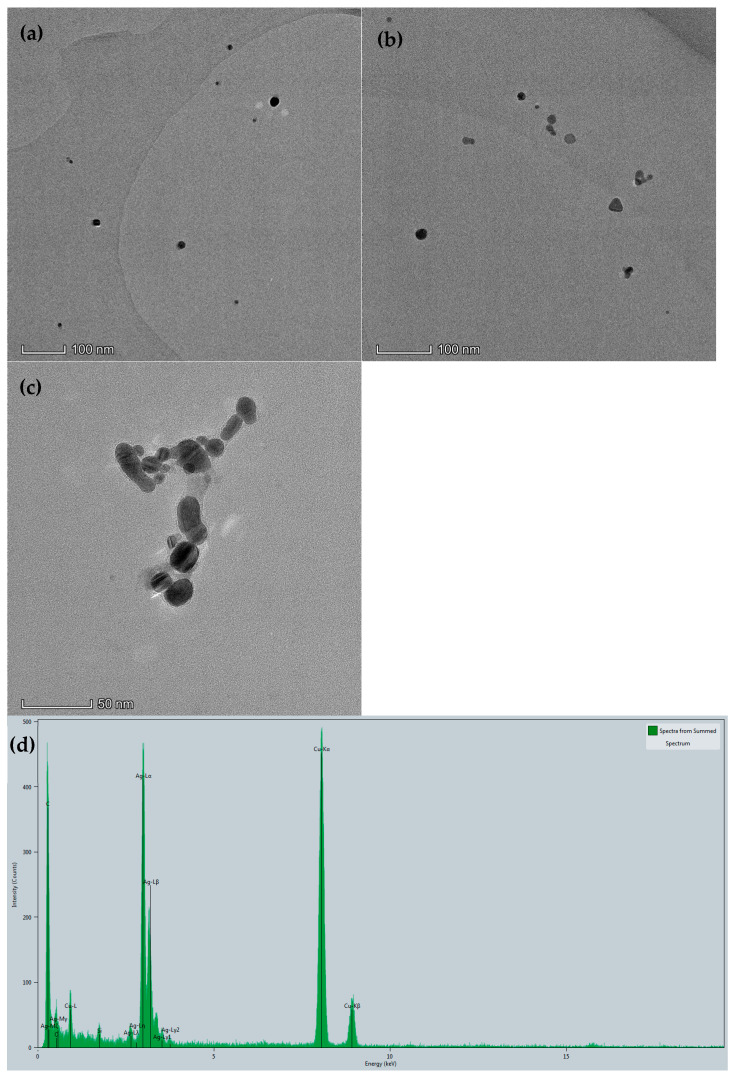
TEM and energy spectra; (**a**) AgNPs; (**b**) 100.00 µmol/L AgNPs + 2.50 nmol/L Apt_Hg_ + 7.50 mmol/L NaCl + 3.25 mmol/L Tris-HCl; (**c**) b + 7.50 µmol/L Hg^2+^; (**d**) energy spectrum of AgNPs.

Figure 2 shows AgNPs and their situation in the analysis system. AgNPs are spherical nanoparticles (Figure 2a) with a particle size ranging from 10 to 20 nm and an average diameter of 17 nm. In the analytical system, the addition of Apt_Hg_ can protect AgNPs and keep the system stable, and the AgNPs have basically no changes (Figure 2b). When Hg^2+^ is added, the Apt_Hg_ reacted specifically with Hg^2+^. At this time, the aptamer can no longer protect the AgNPs, and the AgNPs in the system are aggregated (Figure 2c). The energy spectrum of AgNPs is shown in Figure 2d, and the energy peaks at 0.568, 2.984, and 3.347 KeV correspond to Ag-M, Ag-Lα, and Ag-Lβ, respectively.

### 2.3. Abs Spectra

**Figure 3:**
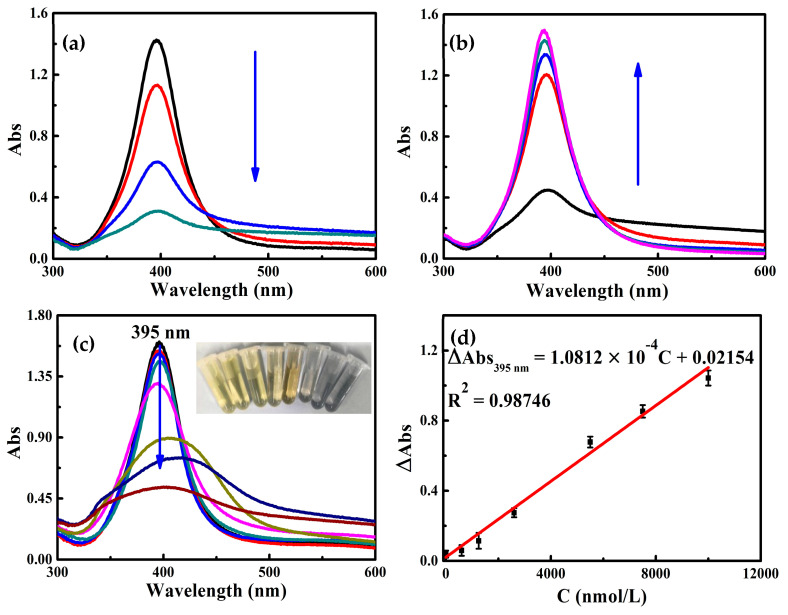
Abs spectra; (**a**) (0, 2.50, 5.00, 7.50) mmol/L NaCl + 100.00 µmol/L AgNPs + 3.25 mmol/L Tris-HCl; (**b**) (0, 2.50, 5.00, 7.25, 12.25) nmol/L Apt_Hg_ + 100.00 µmol/L AgNPs + 3.25 mmol/L Tris-HCl + 7.25 mmol/L NaCl; (**c**) (0, 2.5 × 10^−3^, 0.60, 1.25, 2.60, 5.50, 7.50, 10.00) μmol/L Hg^2+^ + 100.00 µmol/L AgNPs + 2.50 nmol/L Apt_Hg_ + 7.50 mmol/L NaCl + 3.25 mmol/L Tris-HCl (the inset shows the photographs of the color change of the system caused by the increase in mercury ion concentration); (**d**) The standard curve of Abs spectra (**c**).

As the concentration of chloride salts in the system increases, AgNPs will aggregate in a salt solution of a given concentration. The color of the system changed simultaneously. The system color gradually changed from yellow to gray, and the colorimetric signal at 395 nm gradually decreased (Figure 3a). Upon the addition of Apt_Hg_ to the system, the N atoms of the surface bases of the aptamer can coordinate with AgNPs and adsorb on the surface of AgNPs, thereby stabilizing the AgNPs. The color of the reaction solution gradually changed from gray to yellow, and the Abs signal of the system at 395 nm gradually increased (Figure 3b). As the concentration of Hg^2+^ increases, within the concentration range of 2.5 × 10^−3^–10.00 μmol/L, the color of the system gradually changed from yellow to gray, and the Abs signal at 395 nm decreased linearly (Figure 3c,d). According to this, a colorimetric method for Hg^2+^ can be constructed.

### 2.4. Conditional Optimization

**Figure 4:**
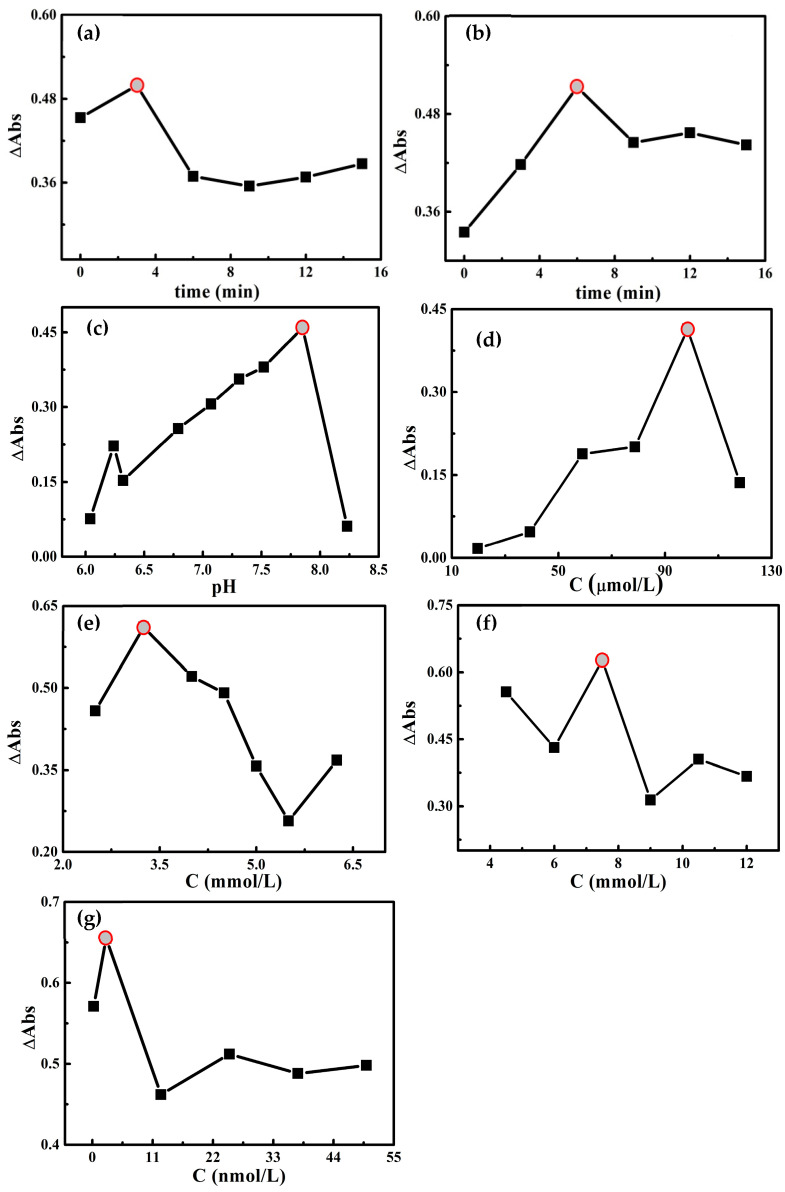
(**a**) The effect of reaction time; (**b**) the effect of standing time; (**c**) the effect of pH; (**d**) the effect of AgNP concentration; (**e**) the effect of Tris-HCl concentration; (**f**) the effect of NaCl concentration; (**g**) the effect of aptamer concentration.

The analysis conditions were optimized by changing the experimental conditions, and the results are shown in Figure 4a–g. When the reaction time was 3 min (Figure 4a), the standing time was 6 min (Figure 4b); the pH was 7.85 (Figure 4c); 100.00 μmol/L AgNPs (Figure 4d), 3.25 mmol/L Tris-HCl at pH 7.85 (Figure 4e), 7.50 mmol/L NaCl (Figure 4f), and 2.50 nmol/L Apt_Hg_ (Figure 4g) were added; the ΔAbs of the system reached its maximum value. Therefore, these conditions were determined as the optimal conditions for this reaction.

### 2.5. Working Curve

Under optimal experimental conditions, the linear relationship between different Hg^2+^ concentrations and their corresponding ΔA was drawn. For the Abs system, within the concentration range of 2.5 × 10^−3^ to 10.00 μmol/L, the change in Abs intensity at 395 nm (ΔAbs_395nm_) exhibited a linear relationship with the concentration of Hg^2+^. The linear equation was ΔAbs_395nm_ = 1.0812 × 10^−4^ C + 0.02154, the linear correlation coefficient R^2^ was 0.98746, and the DL was 2.03 nmol/L (Figure 3d). As shown in Table 1, although some analytical methods have been developed for Hg^2+^ detection, most of them fail to simultaneously integrate sensitivity, simplicity, rapidity, and a wide detection range. Common techniques such as colorimetry, fluorometry, and resonance Rayleigh scattering (RRS) exhibit insufficient sensitivity and narrow detection ranges. While electrochemiluminescence, electrochemical, and surface-enhanced Raman scattering (SERS) methods provide high sensitivity, they require complex operational procedures. The method established by this manuscript is simple to operate, exhibits a wide linear range, achieves a low DL, and shows promising application potential (Table 1).

### 2.6. Effects of Coexisting Interfering Ions

According to experimental methods, the interference of coexisting substances on the determination of 2.50 μmol/L Hg^2+^ by the Abs method was studied. With an allowable error range of ±10%, the following results were observed: 100 times of Cu^2+^, NH_4_^+^, SO_4_^2−^, HPO_4_^2−^, and H_2_PO_4_^2−^; 90 times of K^+^, CH_3_COO^−^; 80 times of NO_3_^−^, Co^2+^, NO_2_^−^, Zn^2+^, SO_3_^2−^, and CO_3_^2−^; 60 times of Fe^3+^; 50 times of P_2_O_7_^4−^, HCO_3_^−^, and Ca^2+^; 40 times of Pb^2+^; 10 times of Mn^4+^ and Cr^6+^; and 5 times of Ba^2+^, Al^3+^ did not interfere with the Abs method for determining Hg^2+^ (Table 2).

As shown in Table 2, most anions and cations do not interfere with the experiment. However, some oxidizing metal ions may destabilize silver nanoparticles, such as Fe^3+^, Mn^4+^, and Cr^6+^. Certain divalent metal cations (e.g., Ba^2+^, Ca^2+^) may compete with mercury ions for binding sites, potentially interfering with the detection. Moreover, hydrolysis products of certain ions (e.g., Al^3+^, Pb^2+^) may interfere with the colorimetric signal of the system, while anions such as P_2_O_7_^4−^ can bind to mercury ions, both potentially compromising the experimental results. For regular water samples, these interfering ions generally pose minimal interference. However, for samples with exceptionally high ion concentrations, pre-treatment strategies (e.g., precipitation or masking agent addition) are advised to eliminate potential analytical interference.

### 2.7. Stability

**Figure 5:**
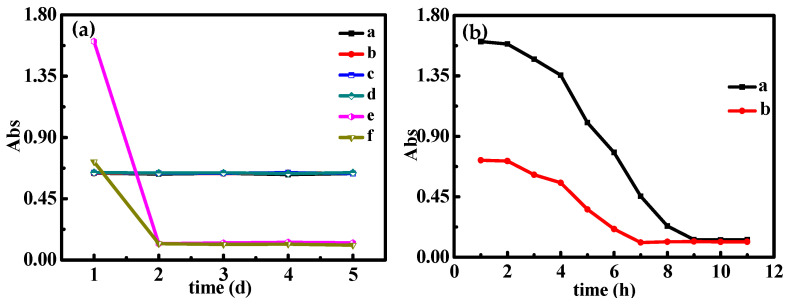
(**a**) The absorbance signal changes in AgNPs stored at room temperature (line a) and 4 °C (line b) (100.00 µmol/L AgNPs). The absorbance signal changes in the analytical platform stored at room temperature (line c) and 4 °C (line d) (100.00 µmol/L AgNPs + 2.50 nmol/L Apt_Hg_). The absorbance signal of the analytical platform changes over 5 days (line e: 100.00 µmol/L AgNPs + 2.50 nmol/L Apt_Hg_ + 7.50 mmol/L NaCl + 3.25 mmol/L Tris-HCl; line f: e + 7.5 µmol/L Hg^2+^). (**b**) The absorbance signal changes of the analytical platform within 11 h (line a: 100.00 µmol/L AgNPs + 2.50 nmol/L Apt_Hg_ + 7.50 mmol/L NaCl + 3.25 mmol/L Tris-HCl; line b: a+7.5 µmol/L Hg^2+^).

The synthesized silver nanoparticles exhibited high stability under both room temperature and refrigerated (4 °C) storage, with absorbance signals remaining essentially unchanged for 5 days (Figure 5a, lines a,b). The analytical platform demonstrated comparable stability, showing negligible absorbance variations under identical storage conditions (Figure 5a, lines c,d). The absorbance signal of the synthesized silver nanoparticles remained essentially unchanged for 5 days under room temperature and refrigerated (4 °C) storage conditions, confirming their high stability (lines a and b in Figure 5a). And the constructed analytical platform (AgNPs + aptamer) also demonstrated excellent stability, exhibiting negligible changes in absorbance over 5 days under room temperature and refrigerated (4 °C) storage conditions (Figure 5a, lines c,d). Time-dependent changes in the colorimetric signal of the analytical platform were systematically monitored. (1) Initial signal stability was maintained during the first 2 h (Figure 5b). (2) A marked signal transition commenced after 2 h, indicating incipient silver nanoparticle aggregation (Figure 5b). (3) The colorimetric signal reached equilibrium after 9 h and remained stable throughout the subsequent 5-day period (Figure 5b; Figure 5a, lines e,f).

### 2.8. Analysis of Real Samples

Water samples collected from three rivers surrounding Guangxi Normal University were utilized as actual test samples for this method. The water samples exhibited a pale yellowish-brown hue without observable precipitates. First, three water samples (10 mL) were accurately pipetted into three 10 mL centrifuge tubes and filtered twice with filter paper to remove suspended solids. Subsequently, water samples were filtered through a 0.45 μm microporous membrane for filtration and centrifuged at 12,000 r/min for 10 min. The supernatant was then extracted and served as the sample test solution. Simultaneously, a reagent blank solution without Hg^2+^ was treated as described above. The samples were tested according to the Abs method, and the results are recorded in Table 3. It is shown by the results that the RSD is below 5.29%, and the recovery rate is between 96.9 to 102.6%, which proves that the method has good stability and accuracy.

## 3. Experimental Section

### 3.1. Instruments and Reagents

The following instruments were used: TU-1901 double-beam UV–vis spectrophotometer (Beijing Puxi General Instrument Co., Ltd., Beijing, China); SK 3300B Ultrasonic Cleaner (Shanghai Kedao Ultrasonic Instrument Co., Ltd., Shanghai, China); FEI Talos F200X Field Emission Transmission Electron Microscope (ThermoFisher Scientific, Waltham, MA, USA); pH meter (Mettler Toledo Instruments Shanghai Co., Ltd., Shanghai, China); KP-26 air energy light-wave furnace (Zhongshan Qiaokang Electric Appliance Manufacturing Co., Ltd., rated power 1200 W, Zhongshan, China); magnetic heating stirrer (79-1, Jiangsu Zhongda Instrument Factory, Changzhou, China); UPW-N series UPW-N15UV ultrapure water machine (Shanghai Xiangfan Instrument Co., Ltd., Shanghai, China).

The following reagents were used: 10 mmol/L AgNO_3_ (Xilong Chemical Co., Ltd., Shantou, China); 0.1 mol/L NaBH_4_ (Sinopharm Chemical Reagent Co., Ltd., Shanghai, China); 0.1 mol/L Tris(hydroxymethyl)aminomethane (Tris, Shanghai yuanye Bio-Technology Co., Ltd., Shanghai, China); 0.1 mol/L HCl (Xilong Chemical Co., Ltd., Shantou, China); Trisodium citrate (Xilong Scientific Co., Ltd., Shantou, China); 30% H_2_O_2_ (Xilong Scientific Co., Ltd., Shantou, China); Hg(NO_3_)_2_·H_2_O (Shanghai Macklin Biochemical Technology Co., Ltd., Shanghai, China); Hg^2+^ aptamer (Apt_Hg_) sequence: 5′-TTTCTTTCTTCCCTTGTTTGTTT-3′ (Sangon Biotech (Shanghai) Co., Ltd., Shanghai, China); 0.1 mol/L NaCl (Xilong Chemical Co., Ltd., Shantou, China). All reagents used were of analytical grade, and no further purification was required. Ultrapure water was used for the experiment.

The preparation of the pH 7.85 Tris-HCl buffer was carried out as follows: 5 mL of 0.1 mol/L Tris solution and 3.45 mL of 0.1 mol/L HCl were added to a 10 mL centrifuge tube, and the volume was diluted to 10 mL to obtain a pH 7.85 Tris-HCl (buffer solution concentration in terms of HCl concentration) buffer solution.

The preparation of orange-red silver nanosol (AgNPs) was carried out as follows: Ultrapure water (44 mL) was added to a conical flask, and 2 mL of 10 mmol/L AgNO_3_, 2 mL of 100 mmol/L trisodium citrate, 600 μL of 30% H_2_O_2_, and 600 μL of 0.1 mol/L NaBH_4_ were added sequentially under stirring. The solution was stirred rapidly until the color turned blue, at which point the blue AgNPs were unstable. The obtained blue nanosilver colloid was immediately transferred to a light-wave stove and heated at 250 °C for 10 min to obtain an orange-red transparent nanosilver colloid. It was naturally cooled to room temperature and then diluted to 50 mL to obtain AgNPs with a concentration of 4 × 10^−4^ mol/L.

### 3.2. Procedure

In total, 500 μL of the prepared nanosilver was pipetted into the 5 mL stoppered test tube, and 50 μL 100 nmol/L Apt_Hg_ was added, shaken, and allowed to stand for 6 min. Then, a certain amount of Hg^2+^, 130 μL of pH 7.85 Tris-HCl, and 150 μL of 0.1 mol/L NaCl were added, shaken, and diluted to 2 mL. After standing for 3 min, the UV absorption spectra were obtained by scanning with a UV-visible spectrophotometer, and the Abs signal of the solution was measured at 395 nm. The solution without Hg^2+^ was used as a blank, the Abs signal was recorded as A_0_, and the value of ΔA = A_0_ − A was calculated.

## 4. Conclusions

In this manuscript, the light-wave method was used to prepare AgNPs nanoprobes exhibiting strong Abs signals. Subsequently, a simple and sensitive label-free aptamer analysis platform for detecting Hg^2+^ was constructed based on AgNPs nanoprobes and label-free aptamers. The constructed Abs analysis platform had a low DL of 2.03 nmol/L and a wide linear range of 2.5 × 10^−3^–10.00 μmol/L. At the same time, this platform was also used for actual sample detection, and it has good stability and selectivity. The constructed Abs analytical platform has potential applications in the detection of Hg^2+^ in the environment. Notably, the analysis platform was established under the conditions of the pH 7.85 Tris-HCl buffer solution and the 7.50 mmol/L NaCl solution. Consequently, applying this method to samples that lack Tris or have different chloride concentrations may alter the speciation of Hg(II), leading to different analytical results.

## Figures and Tables

**Table 1:** Comparison of the reported methods for determination of Hg^2+^.

Method	Linear Range	Detection Limit	Comments	Reference
Electrochemiluminescence sensor	1 × 10^−3^–1 μmol/L	4.71 nmol/L	Complex operation	[33]
Colorimetry	1.0 × 10^−3^–200 μmol/L	0.6748 nmol/L	Poor selectivity, narrow linear range	[34]
SERS	1.0 × 10^−6^–100 μmol/L	2.0871 × 10^−4^ nmol/L	High sensitivity, but complex operation	[35]
Fluorescence	1.0 × 10^−3^–4 × 10^−2^ μmol/L	0.1 nmol/L	Narrow linear range	[36]
RRS	1.0 × 10^−2^–2 μmol/L	4 nmol/L	Low sensitivity and narrow linear range	[37]
Colorimetry electrochemical method	10–60 μg/L 0.001–20 μg/L	3.33 μg/L 3.33 × 10^−4^ μg/L	Poor selectivity	[38]
Abs	2.5 × 10^−3^–10.00 µmol/L	2.03 nmol/L	Wide linear range, easy to operate, and fast	This work

**Table 2:** Effects of coexistent substances on the detection of Hg^2+^ by Abs method.

Interfering Substances	Times	Relative Error	Interfering Substances	Times	Relative Error
Cu^2+^	100	0.74%	SO_3_^2−^	80	3.02%
NH_4_^+^	100	1.23%	CO_3_^2−^	80	−4.56%
K^+^	90	2.52%	P_2_O_7_^4−^	50	2.52%
SO_4_^2−^	100	−0.15%	Pb^2+^	40	3.68%
Co^2+^	80	3.14%	HCO_3_^−^	50	−2.09%
NO_3_^−^	80	3.25%	Ca^2+^	50	0.52%
HPO_4_^2−^	100	1.39%	Mn^4+^	10	4.03%
H_2_PO_4_^2−^	100	0.73%	Cr^6+^	10	−4.53%
CH_3_COO^−^	90	−1.85%	Zn^2+^	80	−2.39%
NO_2−_	80	−2.49%	Ba^2+^	5	−0.49%
Fe^3+^	60	−3.16%	Al^3+^	5	−2.48%

**Table 3:** Measurement results of actual samples.

Samples	Detected Value (μmol/L)	Average (μmol/L) (*n* = 5)	Added (μmol/L)	Found (μmol/L)	Recovery (%)	RSD (%)	Hg^2+^ Value (μmol/L)
1	0.320, 0.312, 0.291, 0.301, 0.316	0.308	2.50	2.730	96.9	3.91	0.308
2	2.868, 2.592, 2.548, 2.653, 2.453	2.623	1.25	3.905	102.6	5.29	2.623
3	2.523, 2.292, 2.262, 2.429, 2.408	2.383	2.50	4.815	97.3	4.46	2.383

## Data Availability

Data will be made available upon request.

## References

[1] Fahim M., Shahzaib A., Nishat N., Jahan A., Bhat T.A., Inam A. (2024). Green Synthesis of Silver Nanoparticles: A Comprehensive Review of Methods, Influencing Factors, and Applications. JCIS Open.

[2] Sani Aliero A., Hasmoni S.H., Haruna A., Isah M., Malek N.A.N.N., Ahmad Zawawi N. (2025). Bibliometric Exploration of Green Synthesized Silver Nanoparticles for Antibacterial Activity. Emerg. Contam..

[3] Thomas S., Gonsalves R.A., Jose J., Zyoud S.H., Prasad A.R., Garvasis J. (2024). Plant-Based Synthesis, Characterization Approaches, Applications and Toxicity of Silver Nanoparticles: A Comprehensive Review. J. Biotechnol..

[4] Yang J., Goksen G., Khan M.R., Ahmad N., Zhang W. (2024). Green-Synthesized Silver Nanoparticles Immobilized on Graphene Oxide for Fruit Preservation in Alginate Films. Food Biosci..

[5] Lieu M.D., Dang T.K.T., Nguyen T.H. (2024). Green Synthesized Silver Nanoparticles, a Sustainable Approach for Fruit and Vegetable Preservation: An Overview. Food Chem. X.

[6] Mishra S., Singh J., Pandey B.K., Dhar R. (2024). Green Synthesis of Oscimum Sanctum Mediated Silver Nanoparticles to Fabricate Sensors for Hydrogen Peroxide Detection. J. Mol. Liq..

[7] Chen F., Liu L., Zhang W., Wu W., Zhao X., Chen N., Zhang M., Guo F., Qin Y. (2021). Visual Determination of Azodicarbonamide in Flour by Label-Free Silver Nanoparticle Colorimetry. Food Chem..

[8] Gu Y., Wu S., Luo Z., Lin L.L., Ye J. (2024). Oppositely-Charged Silver Nanoparticles Enable Selective SERS Molecular Enhancement through Electrostatic Interactions. Spectrochim. Acta A Mol. Biomol. Spectrosc..

[9] Vinayagam R., Nagendran V., Goveas L.C., Narasimhan M.K., Varadavenkatesan T., Chandrasekar N., Selvaraj R. (2024). Structural Characterization of Marine Macroalgae Derived Silver Nanoparticles and Their Colorimetric Sensing of Hydrogen Peroxide. Mater. Chem. Phys..

[10] Zhang D., Chu S., Wang L., Zhan X., Zhou P., Zhang D. (2022). Dual-Mode Colorimetric Determination of As(III) Based on Negatively-Charged Aptamer-Mediated Aggregation of Positively-Charged AuNPs. Anal. Chim. Acta.

[11] Nguyen D.K., Jang C.-H. (2022). Ultrasensitive Colorimetric Detection of Amoxicillin Based on Tris-HCl-Induced Aggregation of Gold Nanoparticles. Anal. Biochem..

[12] Zhao L., Xu J., Xiong L., Wang S., Yu C., Lv J., Lin J.-M. (2023). Recent Development of Chemiluminescence for Bioanalysis. TrAC Trends Anal. Chem..

[13] Duo Y., Xiang Z., Gao G., Luo G., Tang B.Z. (2023). Biomedical Application of Aggregation-Induced Emission Luminogen-Based Fluorescent Sensors. TrAC Trends Anal. Chem..

[14] Wen C.-Y., Liang X., Liu J., Zhao T.-Y., Li X., Zhang Y., Guo G., Zhang Z., Zeng J. (2023). An Achromatic Colorimetric Nanosensor for Sensitive Multiple Pathogen Detection by Coupling Plasmonic Nanoparticles with Magnetic Separation. Talanta.

[15] Li M., Zhang L., Liu W., Jin Y., Li B. (2025). Simple and Low-Cost Colorimetric Method for Quantification of Surface Oxygen Vacancy in Zinc Oxide. Talanta.

[16] Su Y., Yin X., Wei X., Xu R., Wei L., Chen Y., Ding L., Song D. (2025). A Facile Colorimetric Sensor for Ketoprofen Detection in Milk: Integrating Molecularly Imprinted Polymers with Cu-Doped Fe_3_O_4_ Nanozymes. Food Chem..

[17] de Oliveira H.P. (2025). Recent Advances in Colorimetric and Photoluminescent Fibrillar Devices, Photonic Crystals and Carbon Dot-Based Sensors for Mercury (II) Ion Detection. Talanta.

[18] Fu T., Li W., Wen H., Kong L., Zheng M., Ma L., Guo W., Meng Z., Zhang X., Zhang X. (2024). Biocompatibility Evaluation and Imaging Application of a New Fluorescent Chemodosimeter for the Specific Detection of Mercury Ions in Environmental and Biological Samples. Microchem. J..

[19] Nguyen T.H., Sun T., Grattan K.T.V. (2019). A Turn-On Fluorescence-Based Fibre Optic Sensor for the Detection of Mercury. Sensors.

[20] Yuan Y., Wu Y., Wang H., Tong Y., Sheng X., Sun Y., Zhou X., Zhou Q. (2020). Simultaneous Enrichment and Determination of Cadmium and Mercury Ions Using Magnetic PAMAM Dendrimers as the Adsorbents for Magnetic Solid Phase Extraction Coupled with High Performance Liquid Chromatography. J. Hazard. Mater..

[21] Shao Z., Di K., Jia M., Ding L., You F., Wang K. (2025). ZIF-71/MWCNTs Membrane with Good Mechanical Properties and High Selectivity for Simultaneous Removal and Electrochemical Detection of Hg(II). Sep. Purif. Technol..

[22] Vicentino P.D.O., Brum D.M., Cassella R.J. (2015). Development of a Method for Total Hg Determination in Oil Samples by Cold Vapor Atomic Absorption Spectrometry after Its Extraction Induced by Emulsion Breaking. Talanta.

[23] Chen Q., Yao L., Yao B., Meng X., Wu Q., Chen Z., Chen W. (2025). Low-Cost Signal Enhanced Colorimetric and SERS Dual-Mode Paper Sensor for Rapid and Ultrasensitive Screening of Mercury Ions in Tea. Food Chem..

[24] Lesang Madingwane M., Hendricks-Leukes N.R., Tadele Alula M. (2024). Gold Nanoparticles Decorated Magnetic Nanozyme for Colorimetric Detection of Mercury (II) Ions via Enhanced Peroxidase-like Activity. Microchem. J..

[25] Kumar A., Ahmad N., Jadeja Y., Ganesan S., Abd Hamid J., Singh P., Kaur K., Hassen Jaseem L. (2025). Fluorescence Sensor for Mercury Ions in Aqueous Mediums Based on Reduced Graphene Oxide Linked with Molybdenum Disulfide. J. Phys. Chem. Solids.

[26] Wang Y., Yang F., Yang X. (2010). Colorimetric Detection of Mercury(II) Ion Using Unmodified Silver Nanoparticles and Mercury-Specific Oligonucleotides. ACS Appl. Mater. Interfaces.

[27] Chung E., Gao R., Ko J., Choi N., Lim D.W., Lee E.K., Chang S.-I., Choo J. (2013). Trace Analysis of Mercury (II) Ions Using Aptamer-Modified Au/Ag Core–Shell Nanoparticles and SERS Spectroscopy in a Microdroplet Channel. Lab Chip.

[28] Marciniak L., Nowak M., Trojanowska A., Tylkowski B., Jastrzab R. (2020). The Effect of pH on the Size of Silver Nanoparticles Obtained in the Reduction Reaction with Citric and Malic Acids. Materials.

[29] Fernando I., Zhou Y. (2019). Impact of pH on the Stability, Dissolution and Aggregation Kinetics of Silver Nanoparticles. Chemosphere.

[30] Khachornsakkul K., Trakoolwilaiwan T., Leelasattarathkul T. (2026). Distance-Based Paper Microfluidic Analytical Device Using Aptamer Functionalized Silver Nanomaterial for Aflatoxin B1 Quantification in Food Products. Sens. Actuators B Chem..

[31] Malekmohamadi M., Mirzaei S., Rezayan A.H., Abbasi V., Abouei Mehrizi A. (2024). µPAD-Based Colorimetric Nanogold Aptasensor for CRP and IL-6 Detection as Sepsis Biomarkers. Microchem. J..

[32] Ebanks F., Nasrallah H., Garant T.M., McConnell E.M., DeRosa M.C. (2023). Colorimetric Detection of Aflatoxins B1 and M1 Using Aptamers and Gold and Silver Nanoparticles. Adv. Agrochem.

[33] Hu H., Yin Z., Cui H., Wei X., Yu F., Zhang J., Liao F., Wei G., Li Y., Zhang J. (2024). A Novel Dual-Detection Electrochemiluminescence Sensor for the Selective Detection of Hg^2+^ and Zn^2+^: Signal Suppression and Activation Mechanisms. Anal. Chim. Acta.

[34] Guo J., Dong C., Zhang X., Liu Y., Leng Y., Wang G., Chen Z. (2024). Colorimetric Sensors Constructed with One Dimensional PtNi Nanowire and Pt Nanowire Nanozymes for Hg^2+^ Detection. Anal. Chim. Acta.

[35] Park J., Chai K., Kim W., Yoon T., Park H., Kim W., You J., Na S., Park J. (2024). Highly Enhanced Hg^2+^ Detection Using Optimized DNA and a Double Coffee Ring Effect-Based SERS Map. Biosens. Bioelectron..

[36] Che S., Fan Y., Hu X., Yin L., Fu H., She Y. (2025). A Highly Sensitive Fluorescent Probe Based on Functionalised Ionic Liquids for Timely Detection of Trace Hg^2+^ and CH_3_Hg^+^ in Food. Food Chem..

[37] Al-Onazi W.A., Abdel-Lateef M.A. (2022). Catalytic Oxidation of O-Phenylenediamine by Silver Nanoparticles for Resonance Rayleigh Scattering Detection of Mercury (II) in Water Samples. Spectrochim. Acta A Mol. Biomol. Spectrosc..

[38] Diao Q., Bu Z., Feng R., Chen X., Liu J., Tang Z., Liang H., Tian Q., Li S., Niu X. (2025). Performance-Complementary Colorimetric/Electrochemical Bimodal Detection of Hg^2+^ Based on Analyte-Accelerated Peroxidase-Mimicking Activity of GO-AuNPs. Sens. Actuators B Chem..

